# Self-Assembling Organic Micro-/Nano-Pillars on Gold and Glass Surfaces

**DOI:** 10.3390/nano4030768

**Published:** 2014-09-02

**Authors:** Hai-Feng Ji, Wenli Ruan, Yingying Li, Guohua Ding

**Affiliations:** Department of Chemistry, Drexel University, Philadelphia, PA 19104, USA; E-Mails: wr56@drexel.edu (W.R.); liyyl@163.com (Y.L.); dinggh@glut.edu.cn (G.D.)

**Keywords:** self-assembly, organic nanopillars, organic micropillars, vertical nanowires

## Abstract

In this work, we report the formation of a family of organic micro-/nano-pillars prepared from surface-assisted self-assembly processes and factors controlling the growth of the pillars. These acids include cyanuric acid (CA), 1,3,5-benzenetricarboxylic acid (TMA), 1,2,4,5-benzenetetracarboxylic acid (TA) and 3,4,9,10-perylenetetracarboxylic acid (PTA). Aqueous solutions mixed with acids and melamine (M) can be fine-tuned to prepare ordered micro-/nano-pillars on substrates, which can be further optimized for their applications.

## 1. Introduction

For the past forty years, researchers have been pursuing novel materials for high performance electronics and optoelectronics [1,2]. Devices prepared from these nano- and micro-scale structures offer improved photonic and electronic performance over current designs. 1D semiconductor nanostructures, such as nanowires, have been gaining popularity in the research community, due to their ease of synthesis and unique optical, mechanical, electrical and thermal properties [3]. A key barrier to wide-scale integration of functional 1D nanostructures into devices is the difficulty of reproducibly forming efficient electrical contacts between nanostructures and electrodes.

One approach to mass-produce 1D nanowire arrays is to fabricate vertically-oriented nanopillars [4]. The uniqueness and advantage of the nanopillar technique is that the structures are directly connected to the surface by being grown in place, whereas other processes have picked and placed nanowires to make similar connections [5]. Being fabricated *in situ* allows the nanopillar devices to have controllable interconnection of the nanopillar devices between electrodes through a vertical integration process by using no or only relatively coarse lithography.

Significant progress has been made in the past few years in fabricating nanopillars and nanodevices based on nanopillars, with enhanced electronic and optical properties [6,7,8,9,10]. The materials for these nanopillars include silicon, metals, metal oxide, ceramics and polymers [11]. Organic nanopillars, which form in crystals, not amorphous polymers, are rare. Compared to inorganic materials, organic materials are relatively cost-effective and easy to manufacture, and in addition, organic-based devices are continually added to market.

We have recently developed a novel, surface-assisted self-assembling approach to harvest vertically-aligned, single-crystalline and supramolecular nanopillar semiconductors from a solution mixture of cyanuric acid (CA) and melamine (M) [12]. The term, “supramolecular” refers to structures primarily formed by non-covalent interactions, for applications as basic electronic components [13,14]. The nanopillars prepared by this method can be made in large quantities at a low cost due to the facile self-assembling method. This makes the method a viable option for preparing nanopillar-based devices or coatings. The advantages of this type of nanopillars for nano- and opto-electronic applications compared to inorganic ones include a broader range of wavelength for light absorption and emission, scalability and cost-effectiveness, the ease of fabrication, growth on flexible substrates, *etc.*

Although the surface-assisted crystallization method seems straightforward, it is largely unknown what controls the growth of micro-/nano-pillars on surfaces, since this method is in its infancy. In this work, we report the formation of crystalline organic micro-/nano-pillars from three more complexes on gold surfaces by means of the surface-assisted crystallization method. The focus of this work is to summarize factors controlling the formation of the micro-/nano-pillars from these organic building blocks, which is critical for the consequent applications of these pillars.

## 2. Results and Discussion

**Figure 1:**
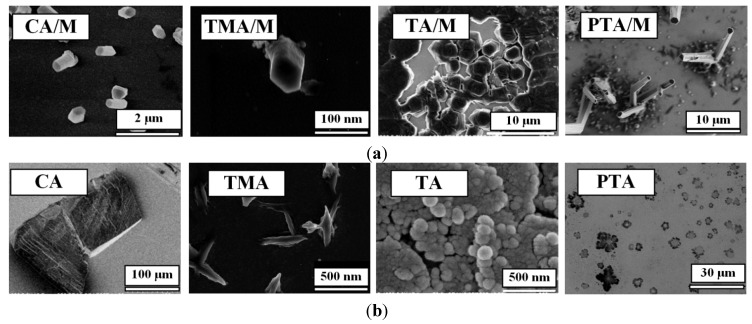
(**a**) Scanning electron microscopy (SEM) images of micro-/nano-pillar arrays prepared from melamine (M) with a series of acids (0.001 M in water) on gold. All of the pillars were prepared from aqueous solutions; (**b**) SEM images of the precipitations of each acid alone after the evaporation of water. CA: cyanuric acid; TMA: trimesic acid; TA: 1,2,4,5-benzenetetracarboxylic acid; and PTA: 3,4,9,10-perylenetetracarboxylic acid.

Via weak interactions, such as hydrogen bonds, π–π stacking, hydrophobic interactions and van der Waals interactions, the molecular assemblies can be hierarchically stacked or coordinated into insoluble nano- or micro-structures in 3D space [12]. We recently reported that [12] when mixing CA and M at low concentrations on a gold surface, an array of nanopillars of the CA/M complex could be obtained (scanning electron microscopy (SEM) images are in Figure 1). In this facile approach, a 20–500 μL acid solution at various concentrations in water was cast onto the gold surface; then, another 20–500 μL of the complementary M at various concentrations in water was added to mix the two chemicals directly onto the surface. Water was allowed to evaporate at ambient temperature, yielding a white coating on the surface. The white coating was made of an array of micro-/nano-pillars of the CA/M complex.

**Figure 2:**
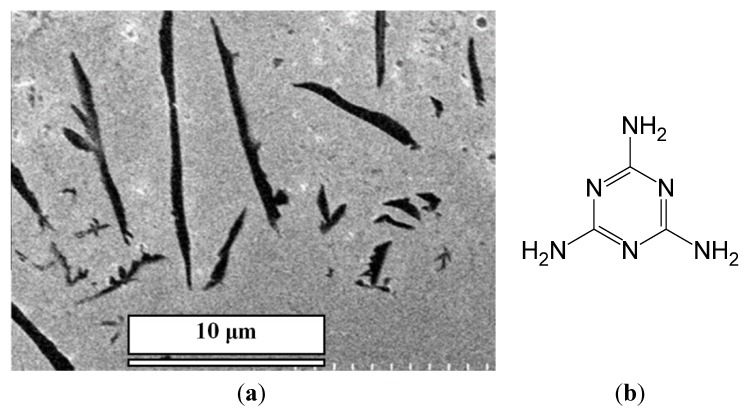
(**a**) SEM images of white precipitation film of M alone after evaporation of water; (**b**) The structure of M.

In this work, by using this approach, we have developed micro-/nano-pillars from three other complexes, including trimesic acid (TMA)/M, 1,2,4,5-benzenetetracarboxylic acid (TA)/M and 3,4,9,10-perylenetetracarboxylic acid (PTA)/M (Figure 1), at micro- and nano-scales on gold surfaces. These acids were chosen, because they are planar molecules, which were expected to self-assemble into a layered structure, leading to the growth of nanopillars. For a comparison, control experiments showed that precipitation of acid molecules alone does not form ordered micro-/nano-pillars on the surface (see Figure 1, bottom row, for direct comparison). Precipitation of M alone does not form nanopillars either (Figure 2).

**Figure 3:**
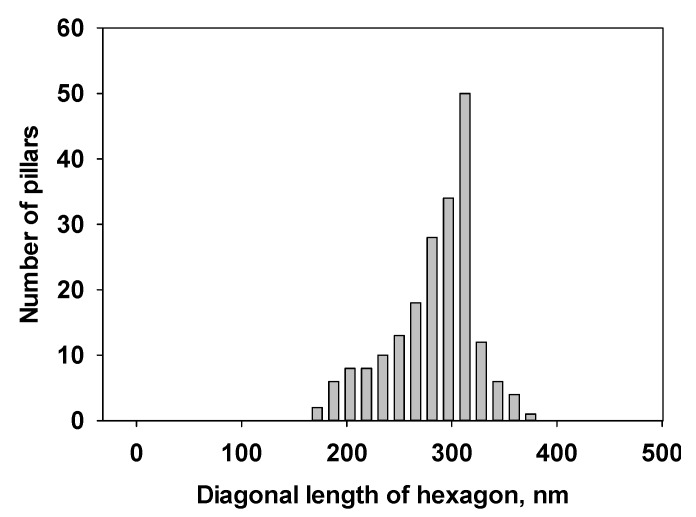
Size distribution of the CA/M pillars prepared from M with CA (both 0.001 M in water) on gold.

All of these systems efficiently produced the epitaxial growth of micro-/nano-pillars on gold substrates. Although the pillars were oriented perpendicular to the plane of the substrate, most of the pillars have variable tilt angles. Typical lengths ranged from 1 μm to 20 μm and diameters from 100 nm to 5 μm. The density of the nanopillars on the gold surface varied depending on the growth time, volume and the concentration of the acids and M. While some trapezoid and rectangular shapes were observed, the most popular morphology of the pillars is hexagonal for all of the acid:M systems. This suggests that the hydrogen networks in these systems are hexagonal, which will be studied by using X-ray diffraction (XRD) in the future. In general, the size of the pillars prepared under the same condition is uniform. Figure 3 shows a size distribution of the CA/M pillars as an example.

The factors controlling the formation and morphology of the nanopillars from these building blocks are summarized as follows.

### 2.1. Substrates

Substrates played a major role in the growth of these micro-/nano-pillars on the substrates, and it even determined the presence or absence of the micro-/nano-pillars. The CA/M micro-/nano-pillars were formed on the gold and glass surfaces, but not on silicon surfaces. On the glass surface, the density of the CA/M nanopillars was much greater, and the length of pillars was shorter than those on the gold surface. The explanation is that the nonpolar silicon surface does not assist the nucleation of these acids and M. On the other hand, the glass surface provided abundant polar sites for faster nucleation of the acid/M complexes than the gold surface, which resulted in smaller nanopillars with a higher density. Most of a gold surface is hydrophobic, but the surface does provide some polar sites for the growth of micropillars. We observed similar phenomena for pillars of other systems in Figure 1.

**Figure 4:**
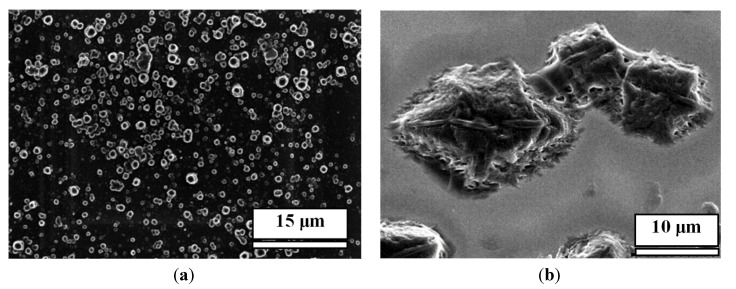
SEM images of CA/M fabricated from 0.001 M CA and 0.001 M melamine on: (**a**) An aminoethanethiol-modified gold surface; (**b**) A dodecylthiol-modified gold surface.

In another set of experiments, we modified the gold surface with monolayers of two alkylthiols in order to understand whether the morphology of pillars can be controlled by the modification of the gold surface. We did not observe micro-/nano-pillars when the gold surface was modified with a hydrophobic monolayer of dodecylthiol, but we did observe nanopillars on a hydrophilic monolayer of aminoethanethiol (Figure 4). This result supported our hypothesis that a nonpolar surface does not assist the nucleation of these acids and M, and the hydrophilic polar surface does provide abundant polar sites for the nucleation of the acid/M complexes.

We concluded that the gold surface was the favored nucleation surface for forming relatively larger micropillars, and the glass surface and amino-monolayer-modified gold surface were appropriate for smaller nano-scale pillars.

### 2.2. Volume of Solutions

**Figure 5:**
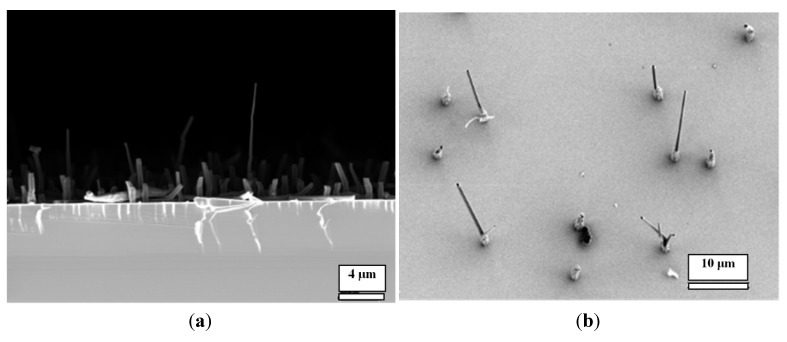
SEM images of micro-/nano-pillar arrays prepared from submerging gold-covered silicon substrates in a 2-mL mixture of M and CA till most of the water was evaporated both: (**a**) 1 mM in water; (**b**) 0.1 mM in water.

In the above experiments, we dropped two solutions on substrates and let the solvent evaporate. If we left the substrate in a mixture solution and let the solvent evaporate slowly, both the diameter and the length of the pillars increases, but the morphologies of the pillars are similar to those prepared from the drop-cast method (Figure 5). The density of the pillars increased as well. A similar result can be achieved by increasing the starting volume, such as 100 μL instead of 20 μL, of both analyte solutions by using the drop-cast method. This makes this surface-assisted method ideal for the tunable growth of micro-/nano-pillars.

### 2.3. Concentration of Analytes

**Figure 6:**
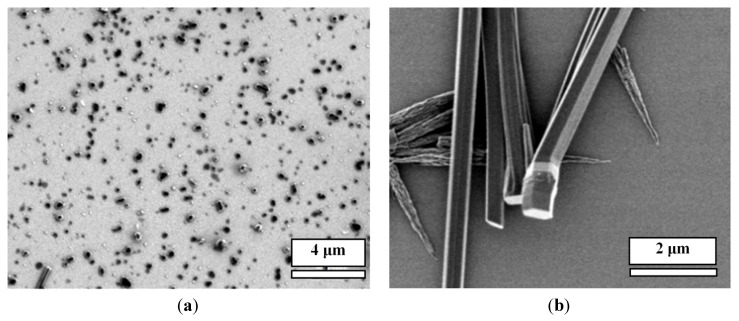
SEM images of: (**a**) CA/M nanoplate on gold surface fabricated from mixing CA and M at 1 × 10^−5^ M on a gold surface; and (**b**) CA/M crystal microwires and the irregular structures on the gold surface fabricated from mixing CA and M at 0.01 M on a gold surface.

The concentration of analytes also has a significant effect on the successful development of micro-/nano-pillars. In all of these systems, concentrations ranging from 1 × 10^−4^ M to 1 × 10^−3^ M were the appropriate concentrations for growing micro-/nano-pillars on gold or glass surfaces. Lower concentrations give only nanoplates-like structures on surfaces (Figure 6a). It was first thought that higher concentrations would yield micro-/nano-pillar arrays at higher density or larger pillars. However, precipitation immediately occurs after mixing, and non-uniform particles and an irregular structure are visible on the surface using SEM, which indicates that nucleation did not initiate on the surface, but rather in solution. As an example, higher concentrations of CA and M result in irregular structures and nano-/micro-wires laying on the surface (Figure 6b). Occasionally, high concentrations yielded both crystal pillars and amorphous structures on glass and gold, but this result could not be reproduced consistently.

### 2.4. pH Effect

**Figure 7:**
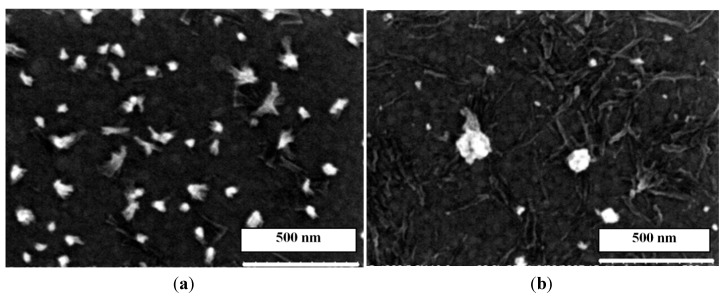
SEM images of TMA/M complexes fabricated on gold substrates from 0.001 M melamine and 0.001 M CA at different pH: (**a**) pH = 5; (**b**) pH = 9.

The pH of the M solutions from 1 × 10^−4^ M to 1 × 10^−3^ M in water was around 8. We prepared a series of the carboxylic acid solutions with various pH adjusted by HCl or NaOH and mixed them with the M solutions. It was found that the acid/M micro-/nano-pillars formed only when the pH was between 6 and 8. The mixing of equivalent amounts of an acid and M at lower pH (such as pH = 5) mostly yielded ill-defined nanostructures on substrate surfaces (Figure 7a), while a higher pH (such as pH = 9) resulted in amorphous structures or films on substrates (Figure 7b).

**Scheme 1:**
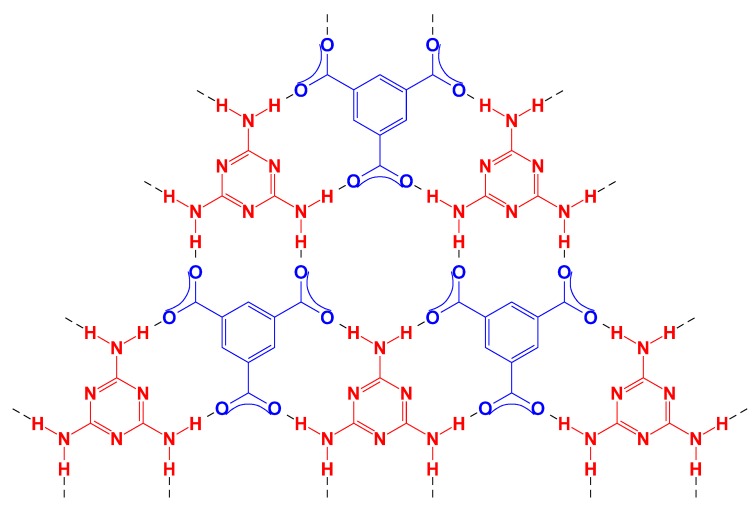
Hydrogen network of TMA and M.

These phenomena may be explained by the dissociation of these organic acids and protonation of M under various pH. The pKa of M is *ca.* 9.1. When the pH is between 6 and 8, >99% of the M is in the electronically neutral state. When the pH is lower, more –NH_2_ is protonated. For instance, 50% of –NH_2_ in M is protonated at pH = 5. Protonation of –NH_2_ affected the planar structure of M, which is needed for hierarchically stacked growth of pillars on substrates [12]. The crystalline structure of the CA/M complex pillars had been reported, which will not be discussed here. The pKa_1_, pKa_2_ and pKa_3_ of TMA are 3.12, 3.89 and 4.70, respectively [15]; the pKa_1_, pKa_2_, pKa_3_ and pKa_4_ of TA are 2.05, 3.25, 4.73 and 6.2, respectively [15]; the pKa of PTA is not known, but expected to be similar to those of TA. The pKa of these acids suggest that when the pH is higher than 9, the majority of the acids are dissociated to carboxylate. For instance, >99.999% of TMA is fully dissociated at pH = 9. Dissociation of carboxylic acids to carboxylates facilitates the formation of a strong hydrogen bonding network of acids and M, which results in the formation of film. Scheme 1 shows a hypothesized hydrogen network of fully-dissociated TMA with M. When the pH is between 6 and 8, both acid and carboxylate moieties exist in the acid molecules; thus, the growth of the hydrogen network is restricted on one plane, resulting in the hierarchical stacking of layers to grow into pillars.

### 2.5. Ratio of Acid and Melamine

**Figure 8:**
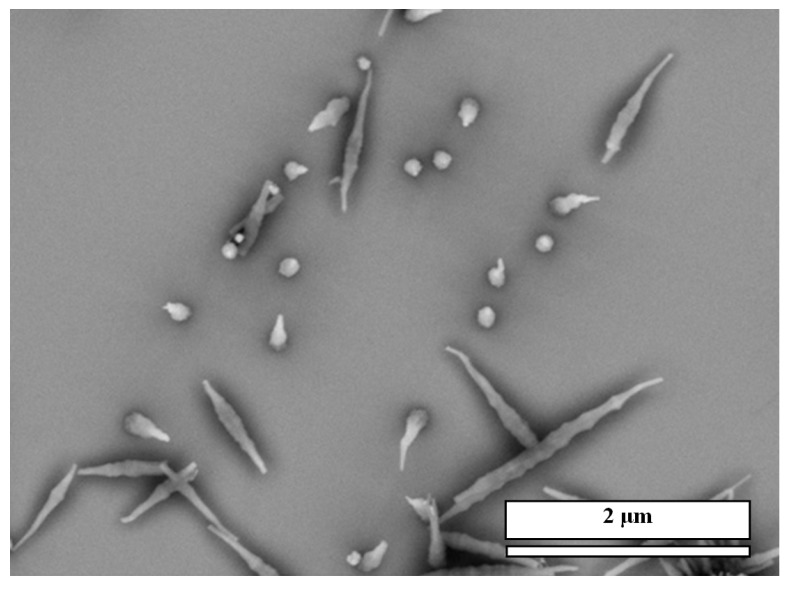
SEM images of CA/M complexes fabricated on gold substrates from 0.001 M melamine and 0.01 M CA at a 1:10 ratio.

The pillars generally formed in a wide range of ratios from 1:10 to 10:1. The ratio of acids and M is not a critical factor for the growth of the pillars. However, the size, density, and morphology of the pillars are affected by the ratios. Detailed studies on each system will be reported in the future. One general rule is that more irregular structures are formed along with crystalline micro-/nano-pillars when the ratio is moved away from 1:1. Figure 8 shows one example of such a phenomenon for which both pillars and irregular structures were formed when the ratio of CA and M is 1:10. This is consistent with the control experiments that acid alone or M alone does not form ordered structures on surfaces.

### 2.6. Mixing Sequence

In an effort to understand the growth of the pillars on surfaces, we investigated the morphology, size and density of micro-/nano-pillars when the two solutions are mixed on substrate surfaces in different sequences, *i.e.*, acids on the surface first, then M is added, or M on the surface first, then acids are added. We found that the density, size and morphology of the micro-/nano-pillars on the gold surface were independent of the mixing sequence of the two analytes.

## 3. Experimental Section

**Scheme 2:**
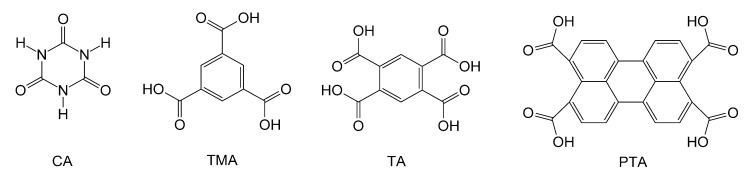
Structures of acids used in this work.

CA (from Alfa Aesar, Ward Hill, MA, USA), 1,3,5-benzenetricarboxylic acid (also called TMA, from Alfa Aesar), TA (from Alfa Aesar), M (Baker Chemical, Center Valley, PA, USA) and PTA (from Luketech, Cherry Hill, NJ, USA) (Scheme 2) were used as purchased. Thin-film gold substrates were prepared by e-beam evaporation. 150 mm-diameter test silicon wafers (Siltronic, Singapore) were coated with a 3-nm thin film of chromium, followed by a 20 nm-thick deposition of gold. Prior to use, substrates were gently rinsed with deionized water and methanol, followed by treatment with ultraviolet (UV)/ozone for 20 min.

SEM was obtained using a Hitachi Tabletop Microscope (TM-1000, Tokyo, Japan).

Experiments were performed using a range of concentrations of solutions for both acids and M in water. The range of concentrations utilized for the experiments were 1 × 10^−2^ M, 3 × 10^−3^ M, 1 × 10^−3^ M, 3 × 10^−4^ M, 1 × 10^−4^ M, 3 × 10^−5^ M, 1 × 10^−5^ M, 3 × 10^−6^ M and 1 × 10^−6^ M, which were prepared by series dilution.

Typically, equimolar amounts of both acids and M were mixed onto gold, silicon and glass substrates with amounts that varied from 20 µL to 500 µL of each solution. After the solvent was completely dry, samples were characterized using SEM.

## 4. Conclusions

In summary, we have developed a novel, yet facile, approach to fabricating organic nanopillar arrays from several molecular building blocks. Factors controlling the formation of pillars are discussed. The size and morphology of the crystalline nano-/micro-pillars can be controlled by the substrate surface, the concentration of analyte, the pH of solutions and the volume of solutions. The surface-assisted crystallization method has been demonstrated to be a viable and possibly common method in developing arrays of organic micro-/nano-pillars. The ability to design optoelectronics using organic nanopillars based on self-assembling processes and integrating them onto various substrates can lead to cost-effective, novel and, in some areas, superior functionalities. The characterization and applications of these nano-/micro-pillars will be investigated and reported in the future.
